# Commentary: Association between triglyceride glucose-body mass index and 365-day mortality in patients with critical coronary heart disease

**DOI:** 10.3389/fendo.2025.1618817

**Published:** 2025-06-25

**Authors:** Yonghai Peng, Ruizi Shi, Decai Wang, Xintao Zeng, Jianjun Wang

**Affiliations:** ^1^ Department of Hepatobiliary Surgery, Mianyang Central Hospital, School of Medicine, University of Electronic Science and Technology of China, Mianyang, China; ^2^ Department of Urology, Mianyang Central Hospital, School of Medicine, University of Electronic Science and Technology of China, Mianyang, China

**Keywords:** triglyceride glucose-body mass index, coronary heart disease, restricted cubic spline analysis, intensive care unit, mortality

## 1 Introduction

We read with great interest the study by Tian et al. (1), in which the research team, using the MIMIC-IV database, explored for the first time the association between the triglyceride glucose-body mass index (TyG-BMI) and 365-day mortality in critically ill patients with coronary heart disease (CHD) admitted to the intensive care unit (ICU). The restricted cubic spline (RCS) analysis demonstrated an L-shaped relationship between the TyG-BMI index and the 365-day mortality rate in this patient population. Furthermore, the authors conducted subgroup analyses based on age, sex, hypertension, type 2 diabetes, type 1 diabetes, chronic kidney disease, acute myocardial infarction, and stroke, to investigate the relationship between the TyG-BMI index and critically ill CHD patients in different subgroups. This study is methodologically sound, with a sufficiently large sample size, and its conclusions provide valuable insights for improving the clinical management of critically ill CHD patients. However, we would like to offer the following points for further discussion regarding the academic value and potential areas for enhancement of this study.

## 2 Multivariable cox regression model covariate adjustment

In the fully adjusted Cox regression model (Model 4), there is a potential methodological issue of multicollinearity that warrants clarification to enhance the robustness of the study’s findings (2, 3). Model 4 simultaneously adjusted for over 20 covariates, accounting for demographic factors, vital signs, and laboratory results, reflecting the comprehensiveness of the statistical analysis. However, certain variables exhibit potential multicollinearity. For instance, the SOFA score already incorporates information on organ dysfunction (such as renal and liver function), which may overlap with variables like creatinine and bilirubin. Additionally, the APSIII score includes information on white blood cell count, systolic blood pressure, and heart rate, which could lead to redundancy. Furthermore, the four critical illness scoring systems—SOFA, APSIII, SAPSII, and OASIS—share overlapping components. For example, both SOFA and SAPSII assess organ dysfunction, while APSIII and OASIS include overlapping indicators, such as heart rate and blood pressure. The collinearity between these variables could inflate standard errors, making hazard ratio estimates unstable and reducing the interpretability of individual predictors. To further optimize model stability and strengthen the robustness of the study’s findings, the authors could consider using variance inflation factor (VIF) testing or clinical relevance-based selection (e.g., retaining the SOFA score while excluding highly overlapping variables).

## 3 Exploration of the optimal threshold for the TyG-BMI index

The authors utilized tertile grouping to reveal the potential association between the TyG-BMI index and the 365-day mortality risk in patients with CHD. However, the results of the RCS analysis confirmed that the relationship between the TyG-BMI index and 365-day mortality risk in CHD patients follows an L-shaped curve, suggesting the existence of a critical threshold for TyG-BMI index, beyond which the mortality risk stabilizes or plateaus. Therefore, incorporating analysis of the inflection point from the RCS curve or determining the optimal threshold using the Receiver Operating Characteristic (ROC) curve (rather than solely relying on tertiles) may be more conducive to rapid clinical risk stratification and offer greater clinical relevance. We encourage the authors to optimize the reanalysis of the TyG-BMI index threshold by utilizing the inflection point from the RCS curve and the ROC curve. Such an improvement would transform the TyG-BMI index from a research metric into an actionable clinical tool, providing direct information for risk stratification and personalized management in critically ill CHD patients.

## 4 Potential value of mortality cause stratification

The authors have thoroughly examined the relationship between the TyG-BMI index and all-cause mortality in critically ill patients with coronary heart disease (CHD). However, due to the limitations of public databases, it is important to note that only all-cause mortality could be studied, and specific causes of death could not be analyzed. While the TyG-BMI index reflects metabolic dysfunction, such as insulin resistance and lipid abnormalities, which could contribute primarily to cardiovascular mortality through the progression of atherosclerosis, leading to fatal arrhythmias or recurrent myocardial infarctions, non-cardiovascular mortality (e.g., sepsis, renal failure) may be influenced by metabolic inflammation or immune suppression. Given this, exploring the association between TyG-BMI and the causes of death, such as cardiovascular versus non-cardiovascular mortality, would require data from sources that provide detailed mortality cause information. Further studies with multi-center cohorts or additional data sources could help elucidate these mechanisms, enabling a more comprehensive understanding of the TyG-BMI index’s prognostic value and its potential for guiding targeted interventions.

## 5 Generalizability and applicability of the study findings

Although the MIMIC-IV database provides rich clinical data, it is limited to critically ill patients at the Beth Israel Deaconess Medical Center. Therefore, the applicability of its conclusions to non-critically ill patients, populations with different healthcare resource settings, or diverse racial groups remains to be validated. For example, metabolic characteristics may vary in Asian populations. Subsequent studies could incorporate multicenter cohorts (e.g., including European and Asian databases) to verify the external validity of the findings and explore the impact of geographic and healthcare intervention differences on predictive performance.

## 6 Discussion

Overall, the study by Tian et al. provides a significant foundation for the application of the TyG-BMI index in prognostic assessment for CHD, with both its methodology and conclusions bearing important clinical and scientific value. However, by optimizing statistical models, refining the determination of the optimal threshold, and further analyzing the causes of death, there is potential to advance the establishment of individualized risk stratification systems for CHD patients. We look forward to engaging in academic discussions with the authors on these important topics.

## References

[1] Tian J Dong Y Xu Z Ke J Xu H . Association between triglyceride glucose-body mass index and 365-day mortality in patients with critical coronary heart disease. Front Endocrinol (Lausanne). (2025) 16:1513898. doi: 10.3389/fendo.2025.1513898 40255500 PMC12006011

[2] Chen F Si Y . Multicollinearity should be considered when interpreting the findings. Neurocrit Care. (2022) 37:606–7. doi: 10.1007/s12028-021-01349-3 34581962

[3] Kim JH . Multicollinearity and misleading statistical results. Korean J Anesthesiol. (2019) 72:558–69. doi: 10.4097/kja.19087 PMC6900425 31304696

